# Effect of the Current-Collecting Carbon Nanotubes Layer on the Properties of the Lead Zirconate Titanate Film for Vibration Sensors

**DOI:** 10.3390/s25020401

**Published:** 2025-01-11

**Authors:** Victor V. Petrov, Victor V. Sysoev, Nikolay N. Rudyk, Yuri N. Varzarev, Andrey V. Nesterenko

**Affiliations:** 1 Institute of Nanotechnologies, Electronics and Equipment Engineering, Southern Federal University, 347922 Taganrog, Russia; nnrudyk@sfedu.ru (N.N.R.); varzarevyuv@sfedu.ru (Y.N.V.); nest@sfedu.ru (A.V.N.); 2 Institute of Physics and Technology, Yuri Gagarin State Technical University of Saratov, 410054 Saratov, Russia

**Keywords:** lead zirconate titanate (PZT), thin films, carbon nanotubes (CNT), vibration sensor

## Abstract

One of the challenging problems in the research and development of vibration sensors relates to the formation of Ohmic contacts for the removal of an electrical signal. In some cases, it is proposed to use arrays of carbon nanotubes (CNTs), which can serve as highly elastic electrode materials for vibration sensors. The purpose of this work is to study the effect of a current-collecting layer of CNTs grown over silicon on the properties of a lead zirconate titanate (PZT) film, which is frequently employed in mechanical vibration sensors or energy harvesters. For the experiments, a vibration sensor mock-up was created with the PZT-CNT-Ni-V-SiO_2_-Si and PZT-CNT-Ni-V-Si structures where an array of vertically oriented CNTs was grown over an oxidized or high-alloyed silicon substrates by plasma chemical deposition from a gas phase. Then, a thin film of PZT was applied to the CNT layer with a high-frequency reactive plasma spraying. For comparison, the PZT film was applied to silicon without a CNT layer (PZT-Si structure). The calculated average value of the piezoelectric module is 112 pm/V for the Ni-PZT-PT-Ni-Si-SiO_2_ sample, and 35 pm/V for PZT-Ni-SiO_2_-Si. It can be seen that the contact realized with the help of CNT ensures more than three times the best efficiency in terms of the piezoelectric module. The value of the piezoelectric module of the vibration sensor with the PZT-CNT-Ni-V-Si structure was 186 pm/V, and the value of the residual polarization was 23.2 µC/cm^2^, which is more than eight and three times, respectively, higher than the values of these properties for the vibration sensor with the PZT-Si structure. It is shown that the vibration sensor can operate in the frequency range of 0.1–10 kHz.

## 1. Introduction

It is known that nowadays all humans live exposed to many mechanical vibrations and electromagnetic radiations, that is, they are surrounded by so-called “scatter” energy. The device to collect scatter energy is frequently called a “harvester”, which allows one to collect and accumulate energy for its subsequent transmission and use by other devices. Attention is now paid more to the research into and improvement of the materials and design of these units [1,2,3,4]. The main materials employed to convert the mechanical energy of motion and vibration into electrical energy are piezo- and ferroelectric ones, primarily based on zinc oxide [5] and lead zirconate titanate Pb(Zr, Ti)O_3_ (PZT) for most applications [6]. Despite a number of technological features in the formation of its physicochemical and electrophysical structure, the high piezoelectric properties of PZT [7] allow it to properly develop harvesters [8], including their films, which are produced by high-frequency reactive plasma sputtering [9,10], the sol–gel method [11], and pulsed laser deposition [12], to name the most frequently applied technology protocols.

The most common design for piezoelectric vibration sensors and energy harvesters is beam-type transducers [7,10,13,14], which are principally used in microelectromechanical structures (MEMS) [15]. The material of the cantilever beam in this design is silicon or metal while the active material is a piezoelectric film. The small dimensions of the entire structure make it possible to reduce the resonant frequency and expand the range of mechanical deformations, thereby increasing the conversion efficiency [16]. It is also possible to use disk [4] and membrane designs [17]. Altogether, taking the nanostructured materials like nanorods, ZnO nanowires, and PZT as an active layer yields options to develop vibration sensors and energy harvesters on a flexible polymer base [8].

One of the major problems in the R&D of vibration sensors and energy harvesters is acquiring Ohmic contacts in order to properly pick up the electrical signal. In some cases, it is proposed to use arrays of carbon nanotubes (CNTs), which can serve as highly elastic electrode materials for specified devices [14,15,18]. It is known that defect-free CNTs or CNTs containing a low concentration of nitrogen as a donor impurity are frequently employed to develop conductive contacts in transistor structures [19]. Furthermore, the greatest prospects are associated with the use of CNTs as a reinforcing filler in composites and the creation of highly elastic electrode materials based on them, including for vibration sensors and energy conversion devices [18]. Thus, the modification of CNTs by applying ZnO to their surface yields options to increase the value of the generated current by 3–6 orders of magnitude [20]. In particular, the average value of the short-circuit current has been observed to be 75 µA/cm^2^ [20]. Furthermore, Sun et al. considered flexible piezoelectric transducers based on composite materials containing multi-walled CNTs in a mixture with ZnO nanoparticles [21]. In their measurements, they found that employing CNT/ZnO composite material remarkably advances the output voltage values from 0.8 V to 7.5 V when compared to pristine CNTs. Yan et al. studied [22] the effect of the percentage of CNTs in mixture with BaTiO_3_ fibers as a piezoelectric composite on the electrophysical, dielectric, and piezoelectric properties of the developed nanogenerator. The maximum value of the output voltage, ca. 3.73 V, current, ca. 1.37 µA, and power, ca. 0.33 μW, were achieved with the ratio of BaTiO_3_/CNT mixture in the composite equal to approx. 40 vol. % and 2 vols. %, respectively. It is worth noting that the generated energy was enough to power at least a single light emitting diode (LED) and recharge the capacitor. Also, it was recently shown that the side walls of CNTs could serve as contacts in piezoelectric converters of mechanical energy based on N-CNTs, which have electrical conductivity with a resistance of 19.28 ± 3.08 kOhm/µm [23,24].

These results allow us to formulate the following state-of-art requirements for the internal architecture of vibration sensors and energy harvesters:

- an array of vertically oriented CNTs should be grown over Si substrates with controlled density and dimensions;
- the gaps between the array elements should be filled with piezoelectric material based on nanocrystalline films;
- as the material of the lower contact layer, it is necessary to employ a metal which can serve also as a catalytic layer in order to grow CNTs;
- the upper contact layer should not ensure the existence of a potential barrier at the piezoelectric material/metal film interface;
- a film of ferroelectric material must be applied conformally to the CNT array.

In this work, CNT arrays were grown on substrates of high-alloyed silicon or oxidized silicon. Prior to growing the CNTs, V-Ni catalytic centers were fabricated on the surface of the beds. PZT film was applied over CNT arrays. The efficiency of converting mechanical vibrations into an electrical signal is advanced via enhancing the effective area of the PZT film where charge carriers are generated as a fundamental pre-requisite for further adjusting the efficient vibration sensors and harvesting units. Therefore, we consider here the influence of a CNT array grown on substrates of high-alloyed Si or Si/SiO_2_ on the piezoelectric properties of PZT films.

## 2. Materials and Methods

CNT arrays were grown on high-alloyed Si substrates (KES-0.001) or oxidized silicon with a pre-applied V layer and a Ni catalytic layer. The silicon substrates underwent conventional clean steps. Metal films were formed by vacuum thermal sputtering using an UVN-2M installation (JSC “Quartz”, Kaliningrad, Russia). The substrate temperature upon the spraying was maintained in the range of 100–250 °C. Herein, to form catalytic centers, a thin film of a buffer (or adhesive) layer was applied to the surface of a silicon substrate prior depositing a layer of catalytic metal. For this purpose, a thin V film, 1–3 nm thick, was utilized. The thickness of the catalytic Ni film was 5–10 nm. In its turn, CNTs were grown by plasma-chemical vapor deposition following three technological steps. CNT growth modes were studied earlier [23,24,25], which allowed us to identify the optimum values for obtaining an ordered array of vertically oriented CNTs. In the first step, the substrate was heated up to a temperature of 660 °C. The pressure was kept constant throughout the entire process at the level of 4.5 Torr. During the heating process, argon and a small amount of ammonia were supplied to the reaction chamber. Argon flow, 40 sccm rate, serves to pre-purge the chamber, displace air, while a minor addition of NH_3_, 15 sccm rate, to the mixture yields a reducing atmosphere in the reactor to prevent the oxidation of the catalytic film. Upon the heating of the substrate to a pre-determined temperature, the metal film coagulates that ensures forming catalytic centers. In the second step, the catalytic centers are activated at a high-frequency discharge plasma in an ammonia flow, 200 sccm rate, for ca. 1 min to remove the oxidized layer from the nickel catalytic centers. It is known that CNTs appear just along the edges of the structure in case the process is conducted without such an activation stage. Furthermore, amorphous carbon is also formed. These features are matured from oxidizing the metal in the catalytic centers at Si substrate upon a heating. As a result, the catalytic ability of these metal nanoparticles is significantly reduced [23]. In the third step, acetylene was added to the vacuum reactor as a carbon source to grow CNTs. Here, the growth was carried out for 15 min. The NH_3_ and acetylene supply rates were 200–250 sccm and 60–80 sccm, respectively. The plasma source power was 40 W [23,24,25].

The grown CNTs were characterized by scanning electron microscopy (SEM) with a Nova NanoLab 600 microscope (FEI, Eindhoven, The Netherlands) at 10 keV. The crystal perfection of the grown CNTs was evaluated by Raman spectroscopy at the Renishaw inVia Reflex facility (Renishaw plc, Wotton-under-Edge, UK) with a wavelength of 532 nm.

PZT films with a Zr:Ti ratio close to 1:1 and a thickness of about 600 nm were deposited on top of a CNT array using high-frequency reactive plasma sputtering in an oxygen atmosphere (0,51 Torr) using a Plasma 80 SE installation (Dana LLC, Voronezh, Russia). In this case, part of the CNT layer was closed from a deposition and was subsequently used as a lower electrical contact. Also, the PZT film was deposited on a Si substrate to be not coated with CNT.

**Figure 1:**
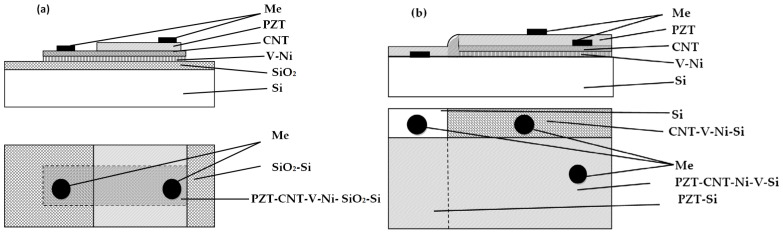
A schematic side view and a top view of (**a**) PZT-CNT-Ni-V-SiO_2_-Si and (**b**) PZT-CNT-Ni-V-Si and PZT-Si structures.

Ni contacts with a diameter of 200 ± 5 µm were formed on top of the PZT layer by vacuum thermal deposition through a shadow mask. The active layers were successively applied to a sample of high-alloyed Si. Upon each application, a part of the sample was shadowed. Thus, PZT-CNT-Ni-V layers were structured on the SiO_2_/Si substrate while PZT-CNT-Ni- and PZT- layers were structured on the Si substrate. A schematic side view and a top view of these structures are provided in Figure 1.

The piezoelectric characteristics of the structures under study were assessed by piezoresponse force microscopy (PFM) using the NTegra probe nanolaboratory (NT-MDT SI, Zelenograd, Russia). The scans were performed upon applying an alternating voltage, of 3 V amplitude and 5 kHz frequency. In the PFM study, we applied the cantilever with conductive Pt coating and a force constant of 11.2 N/m (NSG10 model, TipsNano, Tallinn, Estonia). The value of the piezoelectric module was determined as follows:

$$d_{33}=k\frac{dA}{dU}$$

where *A* is the value of the probe displacement proportional to a vertical piezoelectric response of the sample; *U* is the value of the applied voltage; *k* is the coefficient between the displacement of the probe in nA and the value of the vertical piezoelectric response of the sample in pm; and the *k* coefficient is equal to 22.4 pm/nA for this measuring system. The values of the dielectric constant and residual polarization of the PZT film were obtained from measurements of the capacitance–voltage characteristics of the structures with an Agilent E4980A (Agilent Technologies, Inc. Santa Clara, CA, USA) meter.

**Figure 2:**
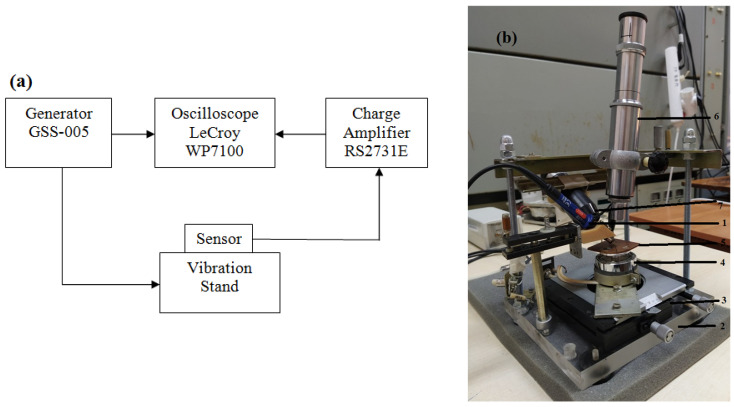
Diagram of the measuring complex (**a**) and the appearance of the vibration stand (**b**) for studying the parameters of vibration sensors (1— ontact probe, 2—base, 3—coordinate table, 4—vibration head, 5—object table, 6—microscope, 7—illuminator).

To study the response of the vibration sensor to mechanical oscillations, a measuring complex was developed as shown in Figure 2. The vibration sensor can have dimensions in the range from 5 × 5 mm^2^ to 30 × 30 mm^2^. Contact pads (Figure 2b, pos. 1) for reading out an electrical signal should be formed with dimensions of at least 100 × 100 μm^2^ over the PZT film (see Figure 1). The base of the stand is a Plexiglas plate (Figure 2b, pos. 2) with dimensions of 160 × 170 × 15 mm. A coordinate table (Figure 2b, pos. 3) is fixed on the base in the center. An AIYIMA vibration speaker (Aiyima JSC, Shenzhen, China), 25 W/4 Ohms, has been placed on the table in the center (Figure 2b, pos. 4). The signal to the vibration speaker is supplied through the BNC connector fixed on the base. The table allows one to move the fixed vibration speaker along the “X” and “Y” axes within 10 mm under 1 μm resolution. A slide table (Figure 2b, pos. 5) made of one-sided foil material is fixed on the vibration dynamics radiator. The copper coating of the table serves as an electrode when placing the sample for a research. When examining a sample, it is possible to carry out a mechanical (vibration) effect with a frequency in the range of 1–10^4^ Hz and read out its electrical response, charge or current.

Because the contact pads fabricated on the surface of the sample are rather small, to be of ca. 0.3 mm in diameter, the stand is equipped with a microscope MIR-2 (Figure 2b, pos. 6) (LOMO JSC, Saint Petersburg, Russia) and an illuminator (Figure 2b, pos. 7) for ease of contact.

A study of the piezo-response of the structures was carried out with the help of a home-made measuring setup. The sample was fixed on a vibrating stand where the mechanical vibrations were excited using a sinusoidal signal from the GSS-005 generator (JSC PriST comp., Moscow, Russia). The electrical signal of the piezoresponse observed in the structures was recorded using a LeCroy WP7100 oscilloscope (LeCroy Corp., Chestnut Ridge, New York, NY, USA).

## 3. Results and Discussion

**Figure 3:**
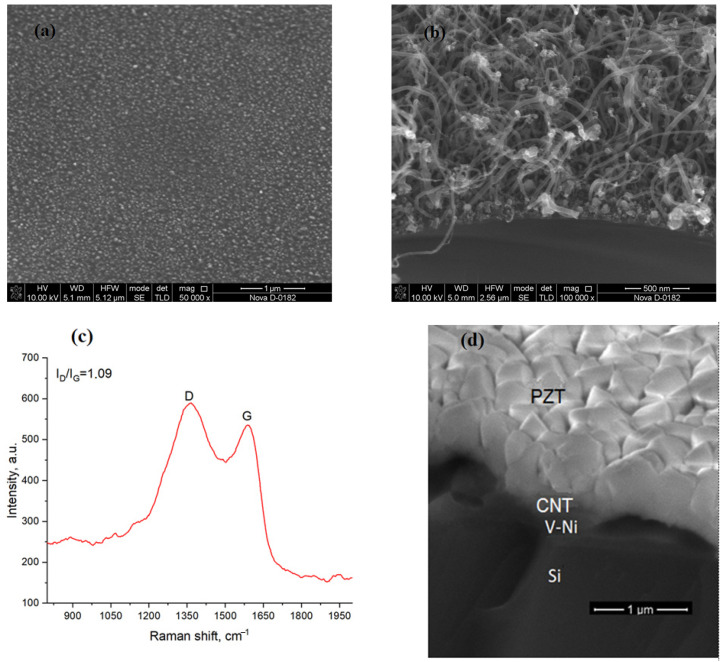
SEM images of catalytic centers formed on a Si substrate heated up to 660 °C (**a**), and CNT array grown over these centers (**b**), Raman spectra of CNTs (**c**), and end face PZT-CNT-Ni-V-Si structure (**d**).

An SEM image of the catalytic centers on a silicon substrate is shown in Figure 3a. The analysis showed that the average height of the catalytic centers was 6.2 ± 2.0 nm, and their diameter was within 290 ± 20 nm. SEM images of the grown CNT array PFM are shown in Figure 3b. It can be seen that CNTs grow to be rather disordered to follow a curved shape. The CNT length is estimated [23,24,25] to be in the range of 0.5–1.0 μm, while the diameter varies from 15 nm to 50 nm, the density of CNT in the array was 10^4^–10^5^ cm^−2^. This shape and size of the CNTs are favorable toward forming an Ohmic contact between high-alloyed silicon and the PZT film.

It was found that the obtained CNTs are multi-walled ones because there is no appearance of radial breathing mode to be characteristic for single-walled CNTs, and the positions of the D-, G-peaks correspond to frequencies ~1363 cm^−1^ and ~1590 cm^−1^ (Figure 3c). The peak intensity ratio I_D_/I_G_ = 1.09 shows a high degree of deficiency in CNTs, which is associated with a violation of the order in the graphite lattice due to the doping of CNTs with nitrogen atoms from ammonia [26]; these defects lead to increasing the intensity of the D-peak.

The SEM image of the PZT-CNT-Ni-V-Si structure is present in Figure 3d which clearly shows that multilayered nature of the entire structure. Previously, we used X-ray diffraction to prove that PZT films appear in a polycrystalline structure with a predominant growth in the (110) direction [9,10,20]. The perovskite phase in the cubic approximation has a unit cell parameter equal to a = 4.10 Å. The broadened peak at 2θ = 31.5° indicates that the crystallite size of the perovskite phase does not exceed 10–20 nm.

**Figure 4:**
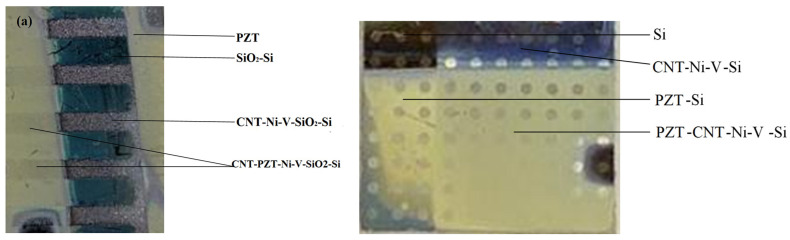
Appearance of samples in PZT-CNT-Ni-V-SiO_2_-Si structure (**a**) and PZT-CNT-Ni-Si, PZT-Si structures (**b**).

Figure 4a shows the appearance of PZT-CNT-Ni-V-SiO_2_-Si sample while Figure 4b presents an image of PZT-CNT-Ni-Si and PZT-Si structures under study. Thus, it was possible to obtain several structures for research at once for a proper comparison.

**Figure 5:**
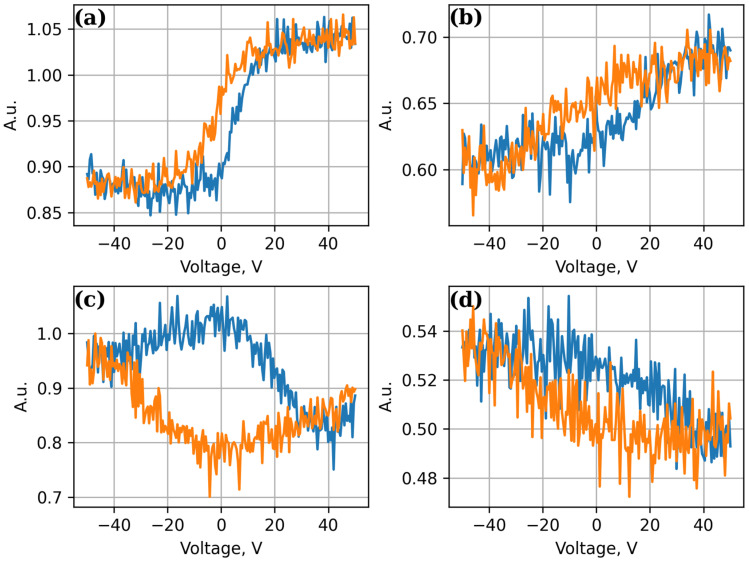
Vertical piezoresponses recorded by the PFM method from the surface of PZT-CNT-Ni-V-SiO-Si (**a**), CNT-Me (**b**), PZT-CNT-Ni-Si (**c**), and PZT-Si (**d**) structures. The blue colored response is the response measured when the electric bias goes from (−) to (+) while red colored response is the one taken vice versa.

Vertical piezoelectric responses recorded by the PFM method from selected protrusions on the surface of PZT-CNT-Ni-V-SiO_2_-Si structures are presented in Figure 5a. In the first case, the measurement of the vertical piezoelectric response was provided by a contact with CNTs that were grown over the Ni-V-SiO_2_/Si structure and a cantilever that was pressed against the selected projection of the PZT film. Several measurements were performed, and the value of the piezoelectric module was calculated for each one. The average calculated value of the piezoelectric module for this sample in contact with CNT is 112 pm/V. Subsequently, the contact was moved to a specially prepared metal contact and the cantilever was pressed against the selected projection of the PZT film. Several measurements were further carried out and the value of the piezoelectric module was calculated for each one. The average calculated value of the piezoelectric module for this sample in metal contact is found to be 35 pm/V. It can be seen that the contact realized with the help of CNT gives more than three times the best efficiency in terms of the piezoelectric module.

Figure 5c displays the vertical piezoelectric responses obtained by the PFM method from selected protrusions on the surface of the PZT-CNT-Ni-V-Si structure. The contact was pressed by the CNT, and the cantilever was pressed against the selected protrusion of the PZT film. Several measurements were performed to test the repeatability of the derived functionality. The average calculated piezoelectric modulus for this structure is 186 pm/V. In the second case (Figure 5b), the structure of PZT-Si was studied. The contact was placed on pristine high-doped Si while the cantilever was placed on PZT film for several repeat measurements. The average calculated value of the piezoelectric modulus was found to be 22 pm/V. These data ensure that the contact formed by the CNTs provides more than eight times better efficiency in terms of the piezoelectric modulus than a contact on pristine silicon.

**Figure 6:**
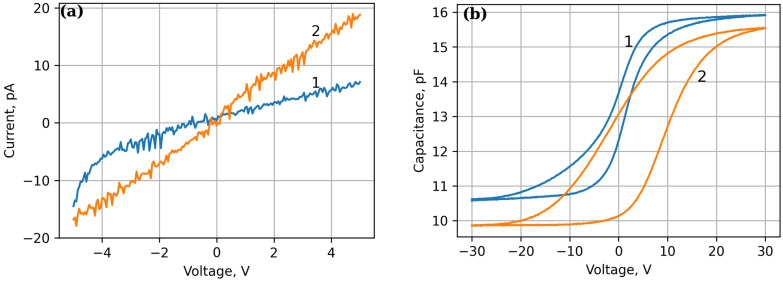
Current-voltage (**a**) and capacitance-voltage (**b**) characteristics of the structures under study: 1—Ni-PZT-Si; 2—Ni-PZT-CNT-Ni-V-Si.

Then, a comparative study of the electrical characteristics of Ni-PZT-Si and Ni-PZT-CNT-Ni-Si structures was also carried out by measuring their current-voltage (I-V) and capacitance–voltage (C-V) curves. We plotted these data in Figure 6. It can be seen that the I-V curve observed in the Ni-PZT-CNT-Ni-Si structure is more linear and shows a higher current than the one in the Ni-PZT-Si structure, indicating that the CNTs provide better ohmic contact. The C-V curves demonstrate a clear pronounced hysteresis which indicates a repolarization of the PZT film. The dielectric constant of the PZT film extracted for both kinds of structures under study was 156.5 ± 0.5. The width of the hysteresis loop measured at the level of flat band capacitance, 15.2 pF for structure Ni-PZT-Si (Figure 6b, curve 1) and 14.3 pF for structure Ni-PZT-CNT-Ni-Si (Figure 6b, curve 2), was 4.3 V and 9.4 V, respectively. The amount of charge generated in the films upon the polarization was calculated according to the method described in [27] to be found equal to 3.43·10^−10^ C and 7.30·10^−10^ C for structures Ni-PZT-Si and Ni-PZT-CNT-Ni-Si, respectively. The residual polarization could be estimated according to these values as 7.6 μC/cm^2^ and 23.2 μC/cm^2^ for structures Ni-PZT-Si and Ni-PZT-CNT-Ni-Si, respectively. These data indicate that the PZT-CNT metamaterial exhibits more than three times higher remnant polarization values than the ones characterizing the PZT film.

**Figure 7:**
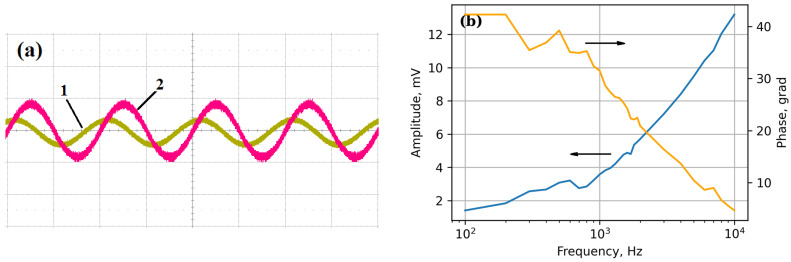
Oscillograms (**a**) of the piezoresponse signal (1) and the exciting signal (2) at a frequency of 1 kHz (Vertical scale—5 mV/div for piezoresponse signal and 1 V/div for the exciting signal; horizontal scale—0.5 ms/div). Frequency response and phase response of the piezoresponse signal (**b**) of the Ni-PZT-CNT-Ni-V-Si structure.

The Ni-PZT-CNT-Ni-V-Si structure was also tested against mechanical vibrations. Figure 7a gives the piezoresponse signal (1) and the exciting signal (2) recorded with an oscilloscope at a frequency of 1 kHz. It can be seen that the sample produces a stable piezoresponse signal to be shifted by a phase relative to the exciting signal. The amplitude of the piezoresponse signal at this frequency is 3.58 mV. When the frequency of the exciting signal changes, the amplitude of the signal and the phase shift of the piezoresponse signal are modified, too. The amplitude-frequency and phase-frequency characteristics measured in the frequency range of 0.1–10 kHz are present in Figure 7b.

## 4. Conclusions

Thus, in this work, it is shown that in an atmosphere of acetylene and ammonia, CNTs doped with nitrogen atoms grow over V-Ni-activated catalytic centers with a diameter of 290 ± 20 nm and a height of 6.2 ± 2.0 nm. CNTs have a length in the range of 0.5–1.0 µm with a diameter varied from 15 nm to 50 nm; the density of CNTs in the array is 10^4^–10^5^ cm^−2^.

A comparative analysis of the electrical characteristics of Ni-PZT-CNT-Ni-Si-SiO_2_, Ni-PZT-CNT-Ni-Si, and Ni-PZT-Si structures showed that the value of the piezoelectric modulus for the structure Ni-PZT-CNT-Ni-Si is 186 pm/V, for the structure Ni-PZT-CNT-Ni-Si-SiO_2_ is 112 pm/V, and the value of the residual polarization was 23.2 µC/cm^2^, which is more than 3–8 times higher than the value of this parameter for the structure without CNT-Ni-PZT-Si. The present results indicate that the use of a CNT layer as an electrical contact to the PZT film readily helps to improve its piezoelectric properties, which is important when manufacturing such electrical units as vibration sensors and energy harvesters. It is shown that the vibration sensor can operate in the frequency range of 0.1–10 kHz.

## Data Availability

The data presented in this study are available on request from the corresponding author.
